# Primary Headache Disorder Among School Students in Kuwait

**DOI:** 10.3389/fneur.2021.621017

**Published:** 2021-02-02

**Authors:** Sameera Shuaibi, Abdelrahman AlAshqar, Samar Farouk Ahmed, Raed Alroughani, Hawraa AlThufairi, Shaikhah Owayed, Fajer AlHamdan, Jasem Al-Hashel

**Affiliations:** ^1^Internal Medicine Department, Ministry of Health, Kuwait, Kuwait; ^2^Obstetrics and Gynecology Department, Kuwait University, Kuwait, Kuwait; ^3^Neurology Department, Ibn Sina Hospital, Safat, Kuwait; ^4^Neuropsychiatry Department, Faculty of Medicine, Minia University, Minia, Egypt; ^5^Division of Neurology, Department of Medicine, Amiri Hospital, Sharq, Kuwait; ^6^Faculty of Medicine, Kuwait University, Safat, Kuwait

**Keywords:** primary headache, prevalence, migraine, tension type headache, school students

## Abstract

**Background:** Primary headaches are remarkably prevalent worldwide and are increasingly reported among children. However, the exact trend in this age group, particularly in the Gulf region, remains largely unknown.

**Aims and Objectives:** To examine the prevalence of primary headache disorders among primary and middle school students in Kuwait.

**Methods:** We conducted a cross-sectional study that included Kuwaiti primary and middle school children and adolescents of both genders in randomly selected schools located in two governorates in Kuwait in the 2018/2019 academic year. Prevalence and attributable burden of headaches, definite and probable migraines, definite and probable tension-type headaches, chronic headaches (≥15 days/month), and probable medication-overuse headaches were assessed using the Headache-Attributed Restriction, Disability, Social Handicap, and Impaired Participation (HARDSHIP) questionnaire for children and adolescents.

**Results:** Of 1,485 questionnaires that were distributed, 1,089 students completed the questionnaire with a respondent rate of 73.4%. The study population consisted of 420 boys (38.56%) and 669 girls (61.43%) students with a mean age of 11.5 ± 2.11 years. The 1-year prevalence of primary headache disorders was 42.78%, with more middle schoolers reporting headaches than primary schoolers (50.37 vs. 30.48%; *p* < 0.02). The mean age of students with primary headaches was 11.98 ± 2.03 years in both genders. When stratified according to diagnostic criteria, migraine headaches were the most frequently reported (20.75%), followed by tension type headaches (18.8%), chronic headaches (2.75%), and probable medication-overuse headaches (0.46%). Primary headaches were significantly higher in girls compared to boys among middle schoolers (66.46 vs. 38.49%; *p* < 0.001); however, no significant difference between genders was noted among primary school students (33.12 vs. 22.33%; *p* < 0.118).

**Conclusion:** Primary headaches are remarkably common in Kuwaiti school students, with migraine headaches being the most frequently reported type. Age and female gender may play a role in the development of primary headaches. These findings necessitate the direction of health services and research efforts toward this age group and warrant the need for further epidemiological studies.

## Introduction

Headache is one of the most common disorders in childhood, with an estimated 75% of children reporting significant headaches by the age of 15 (1). Headaches are defined as a head pain that occurs anywhere in the head or neck areas (2). Headaches amongst children and adolescents are categorized into primary and secondary headaches; the most common primary headaches constitute migraine headaches, tension-type headaches (TTH), and cluster headaches. In children, headaches are more common among the older segment than the younger one and are considered rare prior to the age of 4, with a peak age at 13 years in children (3, 4). Headaches pose a significant burden to both the patient's own quality of life as well as the economy (5–7). In particular, headaches can have remarkable effects on health-related quality of life and school attendance (8, 9). Additionally, children afflicted by headaches are more prone to develop psychiatric disorders, such as depression and anxiety, as well as other somatic symptoms such as abdominal pain (10–12). From an economic perspective, ~50% of the global population is affected by headaches, affecting the economy by £600 million per annum in the UK and between 5.6 and 17.2 billion dollars in the US (13). To date, the exact trend of primary headache prevalence in children is poorly characterized, and early diagnosis and treatment of pediatric patients with headaches are uncommon. Care services for children and adolescents with headaches are yet to meet the needs required, and lack of or late pediatric headache management increases their burden on children and their families. Several studies have addressed the prevalence of pediatric and juvenile headaches, which has indeed been increasing in the last years (14). However, these studies remain scarce worldwide, especially among the pediatric and adolescent age groups; in particular, the Arab world, including Kuwait, are falling short in this regard. Thus, epidemiological studies, including ours, are needed to delineate the magnitude of this debilitating disorder among Kuwaiti children and implement strategies to improve patients' quality of life and ameliorate the financial impact of this disorder.

## Methods

We conducted a cross-sectional study with a school-based sample whereby a questionnaire was distributed to primary and middle school children aged 7–16 years in governmental schools in Kuwait. An equal numbers of boys' and girls' schools were randomly selected from two major governorates in Kuwait: Al-Farwaniyah, the most densely populated with a total of 50,263 students and furthest away from the center of the State of Kuwait, and Hawally, in a more urbanized and central location with a total of 30,909 students in primary and middle schools. These two governorates were chosen to cover the geographic diversity of Kuwait.

### Population of Interest

The study population included Kuwaiti school children aged 7–16 years. Both boys and girls living in Kuwait for at least 1 year prior to the study were enrolled. Only students whose parents agreed to participate in the study and reported that they had no medical history were enrolled in the study.

### Sample Size

According to data from Kuwait's Ministry of Education for the academic year 2018/2019, the number of Kuwaiti students in primary and middle schools was 231,927. Of those, 126,542 (54.56%) were girls and 105,385 (43.44%) were boys. Hence, the sample size was calculated and determined to be 950 students using a special formula based on the reported prevalence of headaches from previous national and international epidemiological studies, which was found to be around 54.4% for headaches and 9.1% for migraines (15, 16). Then, the sample was increased by 20% to overcome the issue of non-response and missing data. Five hundred completed questionnaires were obtained from Al-Farwaniyah governorate (total population of 230,573 residents), whereas 591 completed questionnaires were obtained from Hawally (total population of 221,553 residents). Kuwait's population is complex in its infrastructure, with each governorate varying in terms of peoples' culture, nationality, and background. For example, one governorate may predominate with Bedouin Kuwaitis or “nomads” who are distinct from their fellow urbanized Kuwaitis even in terms of diseases due to consanguineous marriages among first degree cousins, predisposing this population to genetic and other forms of diseases not found in those who do not practice inter-familial marriages. Since governmental schools are purely comprised of Kuwaiti students, we sought not to rely solely on Al-Farwaniyah governorate given its large population, but also to include Hawally to reduce selection bias.

### Survey and Sampling

**Figure 1:**
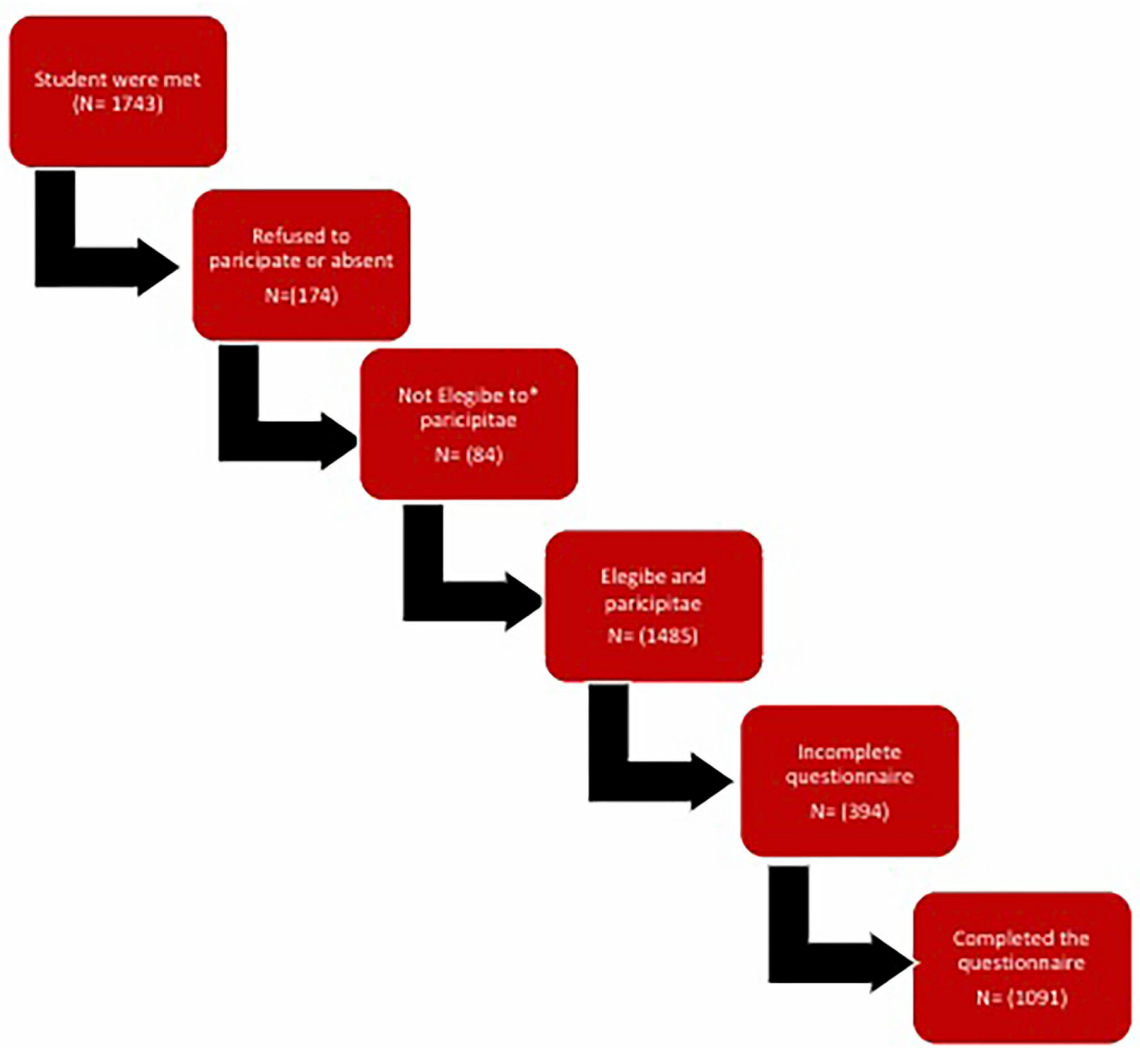
Flow chart of participants. *Non Kuwaiti, Kuwaiti living in Kuwait less than 1 year, has medical or neurological disorder, or Children younger than 7 years.

A representative random sample of school classes was selected stratified by grade (3rd, 5th, 7th, 9th) and school type (male and female schools). The final selection of schools and school classes identified for this study covered an age spectrum from childhood through to adolescence and reflected Kuwaiti students appropriately. All children and adolescents within these selected classes were included, except for those who refused to participate, were non-Kuwaiti nationals, had a history of medical or neurological disease, or were absent on the day of the survey. Of the 1,485 questionnaires that were distributed, 1,089 students completed the questionnaire with a respondent rate of 73.4%. Kuwaiti governmental schools provide free education and thereby only recruit Kuwaiti students, which is purely based on the Kuwaiti regime. As governmental schools are unified in nearly all aspects, we could not resort to privately-owned schools, which operate differently from one another. Figure 1 displays the flow chart of the population selection process.

### Study Tools

The survey used the Lifting the Burden, Headache-Attributed Restriction, Disability, Social Handicap, and Impaired Participation (HARDSHIP) questionnaire that was translated into Arabic (17). The Child HARDSHIP questionnaire for children aged 6–11 years and Adolescent HARDSHIP questionnaire for adolescents aged 12–17 years were used in this study. The HARDSHIP questionnaire has already demonstrated validity and acceptability in multiple languages and cultures, including the Arabic Language. This questionnaire included sociodemographic, screening, and diagnostic questions, as well as questions that pertained to headache burden on quality of life in various domains. The last part of the questionnaire included questions on the utilization of healthcare services in the past, medication use, and a Headache-Attributed Lost Time Index questionnaire. Burden questions addressed the numbers of days missed from school, leaving school early, and experiencing impaired everyday activities due to headaches within the past 4 weeks. Data were obtained from children and adolescents themselves after explanations for the questions were provided by a physician in the study team. Prior to distributing the questionnaires, a brief explanation was provided to clarify the objectives in simple terms. They were assured of the confidentiality of their answers and that human subjects were not to be utilized for the purpose of the study. The students received the questionnaire and discussed it with their parents and, on the second day, well-trained physicians conducted face-to-face interviews and supervised the students while answering the questionnaire. Questionnaire distribution and data collection were organized and conducted by physician supervisors during a school class as a paper-pencil version.

### Diagnosis of Headache

Diagnoses were generated using the HARDSHIP algorithm and applied using the ICHD-3 beta criteria (18, 19). Following the section on sociodemographic data, a question was introduced: “In the last year, have you had a headache that was not part of another illness?” Participants who responded with “no” were classified as headache-free; those who responded with “yes” were asked whether their headache episodes were of one or more types. If the child reported more than one type of headache, subsequent questions addressed the most bothersome headache type. To identify headache type, we asked questions about headache frequency and duration, characteristics, associated symptoms, and use of acute medications. We segregated those reporting headaches for 15 or more days and diagnosed probable medication-overuse headache (pMOH) when acute medications were used on 10 or more days/month. For headaches occurring less than 15 days/month, we applied diagnostic criteria in order to identify definite migraine, definite TTH, probable migraine, and probable TTH. Participants with headache belonging to none of these categories were categorized as “unclassifiable headache.” In the analysis, definite migraine and probable migraine were grouped as migraine, and definite TTH and probable TTH were grouped as TTH.

### Quality Assurance and Validity

The team leader reliably stored all completed questionnaires at the end of each day. Errors were corrected by discussing them with the interviewer and a revisit was arranged if discrepancies could not be corrected. The team leader monitored and assisted researchers on a regular basis to resolve any problems and to review the completed questionnaires.

### Duration of the Project

The fieldwork was carried out over a period of 3 months from 1/10/2018 to 1/1/2019.

### Ethics

The ethical committee of the Ministry of Health and Ministry of Education in Kuwait approved the study. Participants were given a simple explanation about the aim of the study being considered an ethical issue. Written informed consent was obtained from all participants and their parents before the questionnaires were distributed. Students were granted the right to decline participation at any time during data collection. All data were protected in accordance with the ethical guidelines of the Council for International Organizations of Medical Sciences and the principles in the Declaration of Helsinki (20, 21).

### Data Analysis

We used IBM SPSS Statistics 20.0 to conduct data analysis. Data entries were double-checked, with inconsistencies reconciled by reference to the original documents. An error rate of 1.9% was identified. The 1-year prevalence of primary headache disorders was reported as a percentage with 95% CIs. An adjusted prevalence for gender and school grade according to the participation of Kuwaiti students was also reported. Duration of headache was recorded as continuous data in hours. Headache frequency was recorded in days over the preceding 3 months, and typical headache intensity was reported on a verbal rating scale (“not bad,” “quite bad,” and “very bad”). We used proportions, 95% CIs, means, and SDs to summarize the distributions of variables and chi-square for significance of differences. *P* < 0.05 was considered as statistically significant.

## Results

Table 1 demonstrates the sociodemographic characteristics of the study population. A total of 1,089 students participated in the study, of which 420 were primary school students and the remainder were middle school students. The study population consisted of 420 male (38.56%) and 669 female (61.43%) students with the majority being middle schoolers in both genders. The mean age of the participants was 11.5 ± 2.11 years. Collectively, 85.2% of students reported having a headache at least once in their lifetime, whereas 78.09% students had at least one episode in the past year, including primary or secondary headaches, with most episodes being reported by middle schoolers. The 1-year prevalence of primary headache disorders was found to be 42.78% (Table 1). When stratified according to diagnostic criteria, migraine headaches were the most frequently reported (20.75%), followed by TTH (18.8%), chronic headaches (2.75%), and probable medication-overuse headaches (0.46%). Undifferentiated headaches in our cohort were scarce with only seven reported cases (0.6%).

**Table 1:** Demographic data and prevalence of headache among school students.

**Variables**	**Total school students** ***N* = 1,089** **M ± SD/No (%) [CI 95%]**	**Primary school students** ***N* = 420** **M ± SD/No (%) [CI 95%]**	**Middle school students** ***N* = 669** **M ± SD/No (%) [CI 95%]**
**Gender:**			
• Male • Female	420 (38.56%) [35. 83–41.61] 669 (61.43) [58.39–64.17]	103 (24.52%) [20.65–28.86] 317 (75.48%) [71.14–79.35]	317 (47.24%) [43.49–51.03] 352 (52.46%) [48.68–56.21]
Mean Age in yearsRange	11.50 ± 2.11 7–17	9.20 ± 0.10 7–12	12.94 ± 1.13 10–17
Life time prevalence of Any Headache	929 (85.15%) [82.91–87.14]	312 (74.29%) [69.89–78.24]	617 (91.95%) [89.63–93.79]
One year prevalence of Any Headache	852 (78.09%) [75.54–80.45]	271 (64.52%) [59.83–68.95]	581 (86.59%) [83.79–88.97]
One year prevalence of Primary Headache disorder	466 (42.78%) [39.81–45.67]	128 (30.48%) [26.26–35.04]	338 (50.37%) [46.60–54.14]
• **Migraine** • **TTH** • **CH** • **MOH**	226(20.75%) [18.50–23.32] 205 (18.82%) [16.49–21.12] 30 (2.75%) [1.92–3.91] 5 (0.46%) [0.16–1.1]	65 (15.48%) [12.32–19.26] 55 (13.10%) [10.18–16.68] 7 (1.67%) [0.74–3.47] 1 (0.24%) [0.01–1.48]	161 (23.99%) [20.91–27.37] 150 (22.35%) [19.36–25.66] 23 (3.43%) [2.27–5.12] 4 (0.63%) [0.59–0.66]

*TTH, tension type headache; CH, chronic headache; pMOH, probable medication-overuse headache; M, mean; SD, standard deviation; CI 95%, confidence interval 95%*.

Table 2 demonstrates the sociodemographic characteristics of students with headaches. The mean age of students with primary headaches was 11.98 ± 2.03 years in both genders. When stratified according to gender, 51.28% of boys experienced primary headaches as compared to 48.78% of girls. Primary headaches were more frequently reported among male and female middle schoolers. Within the past month, students reported a mean of 6.24 ± 5.18 days of headaches with a mean duration of 9.12 ± 2.51 hours per attack and a mean of 3.26 ± 4.2 days of analgesia use. As regards severity, the majority of students (59.44%) labeled their headaches as “quite bad,” whereas 17.38% of students experienced “very bad” headaches. Headache reporters had to lose a mean of 0.99 ± 2.015 days off school due to headaches and had to leave school early on a mean of 0.77 ± 1.69 days. Table 3 displays the distribution of primary headache disorders stratified by school grade and gender. Table 4 displays the distribution of primary headache disorders stratified by school grade and headache diagnosis.

**Table 2:** Characteristics of Primary Headache among school children with Primary Headache Disorders (No = 466).

**Variables**	**Total school students with primary headache** ***N* = 466** **M ± SD/No (%) [CI 95%]**	**Primary school students with primary headache** ***N* = 128** **M ± SD/No (%) [CI 95%]**	**Middle school students with primary headache** ***N* = 338** **M ± SD/No (%) [CI 95%]**
**Gender:**			
• Male • Female	239 (51.28%) [27.08–35.46] 227 (48.71%) [64.54–72.92]	23 (17.97%) [12.22–25.59] 105 (82.03%) [74.41–87.78]	216 (63.91%) [58.65–68.85] 122 (36.09%) [31.15–41.35]
Mean Age in yearsRange	11.98 ± 2.03 7–17	9.22 ± 0.99 7–12	13.03 ± 1.16 11–17
Number of headache days in the last four weeks	6.24 ± 5.18	5.28 ± 4.82	6.58 ± 5.27
Duration of attacks in hours in the last 4 weeks	9.12 ± 2.51	8.82 ± 2.16	9.32 ± 3.11
Number of analgesic days in the last 4 weeks	3.26 ± 4.20	3.03 ± 3.45	3.35 ± 4.45
Severity of headache			
• Not bad • Quite bad • Very bad	108 (23.18%) [19.57–27.22] 277(59.44%) [54.92–63.81] 81(17.38%) [14.20–21.10]	27 (21.09%) [14.87–29.00] 85 (86.94%) [84.68–88.91] 16 (12.50%) [7.74–19.44]	81 (23.96%) [19.71–28.80] 192 (65.80%) [51.48–61.98] 65 (19.23%) [15.37–23.78]

*M, mean; SD, standard deviation; CI 95%, confidence interval 95%*.

**Table 3:** Character of Primary headache disorders stratified by school stage and gender.

**Gender**	**Male**			**Female**		
**School class**	**Number of headache days in the last 4 weeks** **M ± SD**	**Duration of attacks in hours in the last 4 weeks** **M ± SD**	**Number of analgesic days in the last 4 weeks M ± SD**	**Number of headache days in the last 4 weeks** **M ± SD**	**Duration of attacks in hours in the last 4 weeks** **M ± SD**	**Number of analgesic days in the last 4 weeks** **M ± SD**
Primary school	4.46± 2.50	5.88 ± 2.82	2.54 ± 3.15	5.35 ± 5.08	6.52 ± 3.63	3.13 ± 3.19
Middle school	7.09 ± 5.45	6.88 ± 4.22	3.96 ± 4.20	6.35 ± 5.15	6.64 ± 3.69	3.27 ± 4.10

*TTH, tension type headache; CH, chronic headache; pMOH, probable medication-overuse headache; M, mean, SD, standard deviation*.

**Table 4:** Character of Primary headache disorders stratified by school stage and headache diagnosis.

**Primary headache disorder**	**Migraine**			**TTH**			**CH**			**pMOH**		
	**M** **±** **SD**			**M** **±** **SD**			**M** **±** **SD**			**M** **±** **SD**		
**School class**	**Number of headache days in the last 4 weeks**	**Duration of attacks in hours in the last 4 weeks**	**Number of analgesic days in the last 4 weeks**	**Number of headache days in the last 4 weeks**	**Duration of attacks in hours in the last 4 weeks**	**Number of analgesic days in the last 4 weeks**	**Number of headache days in the last 4 weeks**	**Duration of attacks in hours in the last 4 weeks**	**Number of analgesic days in the last 4 weeks**	**Number of headache days in the last 4 weeks**	**Duration of attacks in hours in the last 4 weeks**	**Number of analgesic days in the last 4 weeks**
Primary school	4.03 ± 2.41	7.84 ± 3.22	2.99 ± 2.59	4.44 ± 2.80	4.07 ± 2.30	2.33 ± 2.48	19.29 ± 5.94	10.57 ± 2.51	6.0 ± 5.06	25	9	20
Middle school	5.59 ± 3.40	9.07 ± 3.50	3.24 ± 3.76	5.28 ± 3.30	3.67 ± 1.76	2.67 ± 3.03	19.91 ± 4.35	9.17 ± 1.99	7.91 ± 3.41	21.25 ± 4.92	12.5 ± 0 2.51	21.25 ± 4.92

*TTH, tension type headache; CH, chronic headache; pMOH, probable medication-overuse headache; M, mean; SD, standard deviation*.

Table 5 demonstrates the 1-year prevalence of primary headache disorders stratified by school grade and gender. Among male students, 22.33% of primary schoolers reported primary headaches as compared to 38.49% of middle schoolers (Table 3). This was similarly observed among females where middle schoolers with primary headaches (66.46%) within the past year were double the number of female primary schoolers with primary headaches (33.12%). The 1-year prevalence of primary headaches was significantly higher among females compared to males among middle schoolers (66.46 vs. 38.49%; *p* < 0.001); however, no significant difference between genders was noted among primary school students (33.12 vs. 22.33%; *p* < 0.118).

**Table 5:** One-year prevalence of Primary headache disorders stratified by school stage and gender.

**Gender**	**Total**			**Male**			**Female**			***P***
**School class**	**Participant students**	**Headache cases**	**Prevalence %(CI 95%)**	**Participant students**	**Headache cases**	**Prevalence %(CI 95%)**	**Participant students**	**Headache cases**	**Prevalence %(CI 95%)**	
Primary school	420	128	30.48% (26.26–35.04)	103	23	22.33% (15.31–31.34)	317	105	33.12 (28.17–38.48)	0.118
Middle school	671	338	50.37% (46.60–54.14)	317	122	38.49% (33.30–43.95)	352	216	66.46% (61.16–71. 38)	0.001*
Total	1091	466	42.71% (39.81–45.67)	422	145	34.36% (29.99–39.02)	669	321	47.98% (44.22–51.77)	0.001*

CI 95%, confidence interval; *significant p-value.

Among primary schoolers, migraine headaches (28.1%) were the most frequently reported in both genders, followed by TTH (24.9%) (Table 6). Female primary schoolers reported all types of primary headaches more frequently than their male counterparts, but no significant difference was noted between genders.

**Table 6:** One-year prevalence of Types of Primary headache disorders stratified by gender in primary school students (*N* = 420).

**Types of primary**	**Male (*N* = 103)**	**Female (*N* = 317)**	***P***
**headache disorder**	**N (%) [CI 95%]**	**N (%) [CI 95%]**	
Migraine	11 (10.7%) [15.31–31.34]	54 (17.04%) [28.17–38.48]	0.120
TTH	13 (12.7%) [33.30–43.95]	42 (13.2%) (61.16–71. 38)	0.674
CH	0	7 (2.2%) [44.22–51.77]	0.128
pMOH	0	1 (0.3%)	0.568

*TTH, tension type headache; CH, chronic headache; pMOH, probable medication-overuse headache; CI 95%, confidence interval 95%*.

Similarly, migraine headaches were the most frequently reported type among female (29.26%) and male (18.18%) middle schoolers (Table 7). This was followed by TTH, chronic headaches, and probable medication-overuse headaches in that order. Female gender among middle-schoolers was significantly associated with a diagnosis of migraine headache (*p* < 0.001) and TTH (*p* < 0.0001).

**Table 7:** One-year prevalence of Types of Primary headache disorders stratified by gender in Middle school students (*N* = 671).

**Types of primary**	**Male (*N* = 319)**	**Female (*N* = 352)**	***P***
**headache disorder**	**N (%) [CI 95%]**	**N (%) [CI 95%]**	
Migraine	58 (18.18%) [14.32–22.80]	103 (29.26%) [24.75–34.22]	0.001*
TTH	52 (16.30%) [12.63–20.77]	98 (27.84%) [23.41–32.75]	0.0001*
CH	10 (3.13%) [1.64–5.75]	13 (3.72%) [3.23–4.23]	0.691
pMOH	2 (0.63%) [0.02–2.41]	2 (0.57%) [0.02–2.19]	0.921

TTH, tension type headache; CH, chronic headache; pMOH, probable medication-overuse headache; CI 95%, confidence interval 95%. *statistically significant.

## Discussion

Our study included Kuwaiti students who had been residents of Kuwait for at least 1 year prior to the survey. Our results demonstrated that a substantial proportion of Kuwaiti primary and middle school children suffered from primary headache disorders. We reported a 1-year prevalence of primary headaches of 42.71% among our study population. This represents a remarkable difference from the results of our previous epidemiological study that demonstrated a 1-year prevalence of 19.4% of primary headache disorders in children and adolescents approached in the community (15). This discrepancy can be contributed to the two different approaches in each study. Children approached in their homes may be influenced by the presence of their parents, who in fact may be the major source of the data, potentially underreporting actual figures. On the other hand, children approached in schools self-report their complaints without any parental influence. Nevertheless, our results are fairly comparable to international studies similarly targeting school children. In Turkey, 46.2% of schoolchildren aged 6–10 years were found to have headaches over a 1-year period (22). These consistent results emphasize the significant magnitude of pediatric headaches, a diagnosis that was once primarily seen to have an adult predilection. Other studies, on the contrary, reported substantially higher or lower proportions of children with headaches. For example, in a study conducted in South Korea (23), 29.1% of school children of both genders reported having a headache over the past year whereas 75.7% of Austrian school children and adolescents aged 10–18 years were found to have headaches over a 1-year period (8). Similarly, 88% of Dutch elementary and secondary school children of both genders reported having a headache at least once over the previous year (4). Many factors could contribute to variations in the reported results, including biological, environmental, cultural, geographical, and those pertaining to methodological differences and diagnostic criteria used in each study. However, the increased prevalence of pediatric headaches can often be explained by improvements in local headache and migraine centers. Headache patients are reviewed by primary care practitioners and patients with suspected secondary headache or refractory headache are referred to secondary or tertiary care. In Kuwait, headache specialists provide continuous education to primary care physicians.

Primary headaches were stratified using diagnostic criteria. Most children were found to have migraine headaches (20.81%), followed by TTH (18.7%), chronic headaches (2.75%), and probable medication-overuse headaches (0.46%). This was similarly documented by other studies where migraine headache was followed in frequency by other headache types (24, 25). In contrast, Rho et al. reported TTH to be the most frequent type among Korean school children, followed by migraine headache (23). Undifferentiated headache in our cohort was scarce with only seven reported cases (0.6%), which is in disagreement with previous reported results in Turkey (25). This discrepancy may be attributed to methodological differences as we reviewed every headache case to confirm an accurate diagnosis.

Our results reported a significantly higher 1-year prevalence of primary headaches among females compared to males. This observation was noted in the overall study population (47.98 vs. 34.36%; *p* < 0.001) and among middle schoolers (66.46 vs. 38.49%; *p* < 0.001) but was rendered nonsignificant among primary schoolers. This was reiterated by Zwart et al. (26), Mehta (24), and our community-based epidemiological study (15), where we found a significantly higher prevalence of headaches among girls. More prevalence of primary headache in girls among middle schoolers could be explained by puberty and female sex hormones (27).

When stratified according to diagnostic criteria, migraines (29.26 vs. 18.18%; *p* < 0.001) and TTH (27.84 vs. 16.3%; *p* < 0.0001) were more significantly reported among female middle schoolers compared to males. However, differences were not statistically significant among primary schoolers and for other types of headaches among middle schoolers. These findings are in line with other epidemiological studies, including our own (15, 17). Laurell et al. detected a higher rate of migraines and TTH among females (28), a finding that was similarly reported by Lyngberg et al. where the male: female ratio for migraines and TTH was 1:6 and 1:3, respectively (29). These findings point to an increased risk of developing primary headaches in females, particularly migraines and TTH, compared to their male counterparts. It has been already established in the literature that women are at an increased risk of developing migraine headaches and are 3.25 more likely to suffer from migraines than men (30). From a pathophysiological perspective, the female brain manifests a sex-specific activity that can be detected via functional neuroimaging. Regions involved in pain modulation and affective processing, such as the insula and precuneus, display enhanced activation in women (31).

On the other hand, it was found that age may play an important role in the development of headaches. Headaches were reported more frequently among middle schoolers compared to primary schoolers in our study (*p* = 0.001), a finding that applied to both genders (32) and was also observed in our previous epidemiological study (15). In further support of our findings, epidemiologic cross-sectional (27, 32, 33) and longitudinal (34) studies show that the prevalence of headaches increases with age. Our findings were in line with an American study that yielded a headache prevalence of 12.84% among 6- to 12-year-old children compared to a prevalence of 21.71% among 12- to 16-year-old children (33). This was also observed in a Canadian study whereby 26.3% of 12- to 13-year-old children and 31.2% of 14- to 15-year-old children experienced headaches (35). Gaßmann et al. (36) reported that 3.6% of 8-year-old children and 10.7% of 15-year-old children had at least one headache weekly. Similar results were mirrored in Germany, Southern England, and Italy (33, 37, 38).

## Strengths and Limitations and of the Study

Our study had several strengths. It is the first of its kind in Kuwait and the Gulf region providing valuable data on the prevalence of primary headache disorders among children and adolescents and their association with gender. Our study represents a great contribution to the regional and global headache epidemiological dataset by closing gaps in the attributable burden of primary headaches in the region. It generated a large randomized stratified sample representative of the entire country. Information obtained from this study may potentially help in predicting the future trends of primary headaches as well as deciding on the optimal management of this disorder in the pediatric population via strategies similar or different from those used in adults.

Our study has limitations inherent to its retrospective design, including recall bias introduced by answering the questionnaire and the reliance on self-reports of clinical parameters of headaches. Being a cross-sectional study, associations between headache and age and gender cannot be interpreted in a causal manner.

## Conclusions

Primary headache disorders are remarkably common in children and adolescents of Kuwait and occur in frequencies comparable to other populations worldwide. Migraine is the most prevalent type in both genders. Female adolescents have a significantly higher risk of developing migraines and TTH compared to their male counterparts, a finding that was not observed among primary schoolers or with other types of headaches. Enhanced understanding of the prevalence of this debilitating disorder among children may allow for proper use of healthcare resources to reduce the burden of this disease, improve the quality of life of patients and their families, and direct research efforts toward delineating the molecular mechanisms of pediatric headaches.

## Data Availability Statement

The raw data supporting the conclusions of this article will be made available by the authors, without undue reservation.

## Ethics Statement

The studies involving human participants were reviewed and approved by the Ethical Committee of the Ministry of Health, Kuwait. Written informed consent to participate in this study was provided by the participants' legal guardian/next of kin.

## Author Contributions

JA-H designed the study and reviewed the manuscript. SA designed the study, performed statistical analysis, and criticized and reviewed the manuscript. AA, SS, HA, SO, and FA performed data collection, data entry, and drafted the manuscript. RA reviewed the manuscript. All authors read and approved the final manuscript.

## Conflict of Interest

The authors declare that the research was conducted in the absence of any commercial or financial relationships that could be construed as a potential conflict of interest.

## Abbreviations

CH: Chronic headache
HARDSHIP: Headache-Attributed Restriction, Disability, Social Handicap and Impaired Participation
ICHD-III: International Classification of Headache Disorders III
pMOH: Probable Medication over use headache
TTH: Tension Type Headache.
